# Microglial histone H3K18 crotonylation promotes STAT1 expression and induces cognitive deficit in Alzheimer disease

**DOI:** 10.3389/fimmu.2026.1744375

**Published:** 2026-01-27

**Authors:** Ying Weng, Ting He, Mengzhu Li, Qiuzhi Zhou, Jiazhao Xie, Linyu Wei, Xin Wang, Jian-Zhi Wang, Maolin Zhong, Shihong Li

**Affiliations:** 1 Department of Pathophysiology, School of Basic Medicine, Huazhong University of Science and Technology, Wuhan, China; 2 Key Laboratory of Education Ministry of China/Hubei Province for Neurological Disorders, Tongji Medical College, Wuhan, China; 3 Department of Anesthesiology, Ganzhou Key Laboratory of Anesthesiology, The First Affiliated Hospital of Gannan Medical University, Ganzhou, China

**Keywords:** crotonic acid, histone crotonylation, microglia, neuroinflammation, STAT1

## Abstract

**Background:**

Alzheimer's disease (AD) is the most prevalent neurodegenerative disorder, yet the epigenetic mechanisms underlying its pathogenesis remain incompletely understood. Histone crotonylation, a novel post-translational modification, has been implicated in neuroinflammation. However, its role in AD-related cognitive impairment has not been elucidated.

**Methods:**

Histone crotonylation was examined in 5xFAD and Aβ42-injected mice. Crotonic acid was administered intracerebroventricular (ICV) to elevate hippocampal histone crotonylation in wild-type mice. Cognitive function was assessed using behavioral tests. Synaptic integrity was evaluated via western blotting and Golgi staining. Microglial activation and co-localization of H3K18cr were determined by immunofluorescence. Transcriptomic analysis identified differentially expressed genes and enriched pathways. The role of signal transducer and activator of transcription 1 (STAT1) was validated in BV2 microglial cells using the STAT1 inhibitor fludarabine.

**Results:**

Hippocampal pan-histone H3 crotonylation (H3Kcr) and H3K18cr were significantly upregulated in both 5xFAD and Aβ42-injected mice compared to controls. ICV injection of crotonic acid markedly elevated hippocampal H3Kcr and H3K18cr levels and induced significant cognitive deficits, shown by impaired novel object recognition and fear conditioning performance. Crotonic acid treatment resulted in synaptic dysfunction, including reduced synaptic markers (SYN1, SYT, GluA2, GluN2B) and decreased CA1 dendritic spine density. Crotonic acid also induced microgliosis with elevated Iba1 expression. H3K18cr was specifically upregulated in microglia, with no significant changes observed in neurons or astrocytes. Transcriptomic analysis identified 478 differentially expressed genes enriched predominantly in immune-related pathways, with STAT1 highlighted as a key upstream transcription factor. In BV2 cells, crotonic acid significantly increased total and phosphorylated STAT1 (Tyr701) levels via a JAK1-independent mechanism. Treatment with fludarabine effectively suppressed STAT1 expression and attenuated the production of pro-inflammatory cytokines, including TNF-α, IL-6, and IL-1β.

**Conclusion:**

This study provides the first evidence that elevated microglial H3K18cr contributes to AD-related cognitive impairment by promoting STAT1 expression and subsequent neuroinflammation. These findings identify microglial histone crotonylation as a novel epigenetic mechanism in AD pathogenesis and suggest that targeting the H3K18cr-STAT1 axis may represent a potential therapeutic strategy for AD.

## 1 Introduction

Alzheimer’s disease (AD) is the most prevalent neurodegenerative disorder, clinically characterized by progressive cognitive decline (1). Although familial AD caused by mutations in genes such as amyloid precursor protein (APP), presenilin 1 (PSEN1), and presenilin 2 (PSEN2) has provided valuable insights into AD pathogenesis, it accounts for only 1% of AD cases (2). The vast majority of AD cases are sporadic and influenced by multifactorial contributors, including aging, lifestyle, environmental exposures, and genetic predisposition (3, 4). Intriguingly, individuals with similar genetic risk factors often exhibit substantial variability in susceptibility, pathological severity, and clinical presentation, pointing to epigenetic mechanisms as a likely driver of this heterogeneity.

Epigenetic regulation, unlike genomic alterations, refers to heritable changes in gene expression that do not involve modifications to the nucleotide sequence. These mechanisms include post-translational modifications (PTMs) of histones, such as methylation, acetylation, phosphorylation, ubiquitination, and crotonylation, which principally regulate gene transcription by altering chromatin structure (5, 6). As a key form of epigenetic regulation, histone modifications not only contribute to neuronal development but also play pivotal roles in aging and AD progression (7). Notably, significant alterations in histone phosphorylation, acetylation, and methylation have been observed in both the brains and peripheral blood of AD patients, further supporting their involvement in AD pathogenesis (8–10).

Among these modifications, histone crotonylation, a recently identified PTM, is evolutionarily conserved and shares similarities with histone acetylation (11). Emerging evidence indicates that histone crotonylation is involved in diverse physiological processes, including gene expression, DNA repair, and cell cycle regulation, as well as pathological conditions such as cardiac hypertrophy, cancers, and neuroinflammation (12, 13). The regulation of crotonylation involves various histone acyltransferases and deacyltransferases, many of which also regulate other PTMs like acetylation, highlighting the interconnected nature of histone modifications (14).

The crotonyl group required for histone crotonylation is provided by crotonyl-CoA, which is closely tied to cellular metabolism. The crotonyl group required for crotonylation is derived from crotonyl-CoA, a metabolite closely linked to cellular metabolism. Exogenous supplementation with crotonate has been shown to elevate crotonyl-CoA levels, thereby enhancing histone crotonylation (15, 16). Among these, H3K18cr exhibits tissue specificity and is highly expressed in the colon and brain of wild-type mice (17).

Microglia, the resident immune cells of the central nervous system (CNS), play critical roles in neurodevelopment, synaptogenesis, homeostasis, and aging (18, 19). In a healthy brain, microglia continuously surveil their environment, but upon encountering infections, trauma, or other insults, they become activated, transitioning into either pro-inflammatory or anti-inflammatory phenotypes depending on the context (20, 21). Excessive or prolonged microglial activation, such as that triggered by misfolded proteins, contributes to neuroinflammation and exacerbates neurodegenerative diseases, including AD (22, 23). Genome-wide association studies (GWAS) have revealed that microglia harbor more AD-associated risk genes than any other neural cell type, and epigenomic studies have identified distinct epigenetic changes in microglia in AD (24, 25). Recent studies have demonstrated that upregulation of histone crotonylation increases pro-inflammatory cytokines such as TNF-α, IL-1β, and IL-6, thereby contributing to neuroinflammation (26).

This study presents the first observation of elevated histone crotonylation levels in the brains of AD mouse models. Furthermore, it investigates the effect of crotonic acid on AD-like cognitive impairment, aiming to determine whether crotonic acid-induced histone crotonylation triggers AD-related pathological damage both *in vivo* and *in vitro*.

## 2 Materials and methods

### 2.1 Animals

5xFAD mice (B6SJL-Tg(APPSwFlLon, PSEN1*M146L*L286V) 6799Vas/Mmjax) were obtained from the Jackson Laboratory, while C57BL/6 mice were acquired from Weitong Lihua Limited Company (Beijing, China). Wild-type controls for 5xFAD experiments were non-transgenic littermates. For other experiments, age-matched C57BL/6J mice from our colony were used. These animals were housed under typical laboratory conditions with a 12-h alternating light/dark cycle, with food and water provided ad libitum. Experiments exclusively utilized male specimens weighing 20–30 g. For histone crotonylation detection, 8-month-old 5xFAD mice and age-matched C57BL/6 mice were used. Additionally, 2-month-old C57BL/6 mice were utilized for intracerebroventricular injections of Aβ42 and crotonic acid. All animal experiments were conducted in accordance with the guidelines of the Animal Care and Use Committee of Huazhong University of Science and Technology.

### 2.2 Stereotaxic injection

Mice weighing 20–30 g were anesthetized with isoflurane (3-4% for induction, 1-2% for maintenance). The scalp was sterilized sequentially with iodophor and 75% ethanol, and then incised along the midline of the skull. Bilateral holes were drilled at the following coordinates: 0.22 mm posterior to bregma, ± 0.9 mm lateral to the sagittal suture, and 2.3 mm in depth. Aβ42 (2 μg/μl, 4 μl, H1368, Bachem, Switzerland) or ACSF (4 μl) (Solarbio, China) was bilaterally injected into the lateral ventricles. Histone crotonylation analysis was performed two weeks after Aβ42 injection. For additional experiments, crotonic acid (800 mM, 2 μl, Sigma-Aldrich, USA) or 0.9% sterile physiological saline was bilaterally injected into the lateral ventricles. Behavioral analysis was conducted at 7 days after the injection of crotonic acid.

### 2.3 Behavioral test

To minimize bias, mice were randomly allocated to either the treatment group or the vehicle-treated control group (n = 8 per group). The solution was prepared and coded by an independent researcher, ensuring objectivity. All behavioral experiments were conducted and analyzed by an experimenter blinded to the treatment codes of the animals.

#### 2.3.1 Object recognition test

The object recognition test relies on the spontaneous preference of rodents for novelty and their ability to recognize familiar objects. Prior to training, mice were habituated to an open-field arena (40 cm × 40 cm × 40 cm) by allowing them to explore the space for 10 minutes, and locomotor activity was evaluated. On the following day, two identical objects were placed in the center of the arena, and each mouse was allowed to explore them for 10 minutes. The exploration time for each object was recorded. For the testing phase, conducted 24 hours after training, one of the objects was replaced with a novel object, while the location remained unchanged. Mice were allowed to explore for 10 minutes, and the exploration time for each object was recorded. The objects used as “familiar” and “novel” during the training and test phases were fully counterbalanced across all animals and experimental groups. Each specific object served equally often in both roles to eliminate any potential bias from intrinsic object properties. The same arena was cleaned with 75% ethanol between trials to eliminate olfactory cues. All sessions were recorded and analyzed using ANY-maze software (O’Hara & Co., Ltd, Japan). The discrimination index (D.I.) for the novel object was calculated using the formula: D.I. = [(Tnovel- Tfamiliar)/(Tnovel + Tfamiliar)] × 100%.

#### 2.3.2 Elevated plus maze

The elevated plus maze consisted of two closed arms (25 cm × 5 cm × 15 cm), two open arms (25 cm × 5 cm × 0.5 cm), and a central platform (5 cm × 5 cm). The maze was elevated 50 cm above the floor. At the start of each trial, mice were individually placed in the central area and given 5 minutes to navigate the maze freely. The duration spent in the open arms was recorded using a video tracking system (Chengdu Taimeng Software Co., Ltd, China).

#### 2.3.3 Contextual fear conditioning

The contextual fear conditioning (CFC) test was conducted as previously described (27). During the training phase, each mouse was placed in a chamber (23 cm × 23 cm × 30 cm) equipped with a metal wire floor and transparent plastic walls, housed within a white soundproof cubicle. After a 3-minute habituation period, mice were subjected to three mild foot shocks (0.7 mA for 2 seconds, with a 60-second interval between shocks). The chamber was cleaned with 75% elthanol after each trial to eliminate residual odors. To assess contextual memory, mice were returned to the training chamber 24 hours later and observed for 3 minutes without receiving an electric foot shock. Freezing behavior, defined as the complete absence of movement except for respiration, and overall activity were recorded and analyzed using ANY-maze software (O’Hara & Co., Ltd, Japan).

### 2.4 Golgi staining

Golgi staining was performed using the FD Rapid GolgiStain Kit, following the protocol of manufacturers (PK401, FD NeuroTechnologies Inc). Spine density was analyzed on secondary and tertiary branches of both apical and basal dendrites in the CA1 region of the hippocampus. A total of 20 segments of either secondary or tertiary dendrites were randomly selected for analysis. The length of the selected dendrites segments was measured using ImageJ software.

### 2.5 Nissl staining

To evaluate neuronal survival, Nissl staining was performed on 30 μm-thick brain sections using cresyl violet. Briefly, free-floating sections were mounted onto gelatin-coated glass slides and air-dried overnight. The slides were subsequently dehydrated through a graded ethanol series (70%, 95%, and 100%, 2 minutes each) and stained in a 0.1% cresyl violet solution (Sigma-Aldrich, C5042) for 15 minutes at 56 °C. Following staining, the sections were briefly rinsed in distilled water and differentiated in 95% ethanol until the desired contrast was achieved, with Nissl bodies appearing dark purple against a light background. The differentiation process was carefully monitored under a light microscope. After differentiation, the sections were fully dehydrated in two changes of absolute ethanol (5 minutes each), cleared in two changes of xylene (10 minutes each), and coverslipped using DPX mounting medium (Sigma-Aldrich). Stained sections were examined under a bright-field microscope (Nikon Eclipse 80i). Viable neurons were identified by their large, clear, round or polygonal cell bodies with distinct nuclei, nucleoli, and intact Nissl bodies within the cytoplasm. In contrast, pyknotic neurons were characterized by shrunken, darkly stained nuclei and fragmented cytoplasm.

### 2.6 Immunofluorescence

Immunofluorescence was performed as previously described (28). Briefly, mice were anesthetized and intracardially perfused with PBS (0.01 M, PH 7.4), followed by 4% paraformaldehyde (PFA) solution (in 0.01 M PBS, pH 7.4). The brains were removed, post-fixed in 4% PFA for 12 hours, and subsequently cryoprotected in 20% sucrose/PBS and 30% sucrose/PBS at 4 °C until the brains sank to the bottom of the solution. Brain sections were cut at a thickness of 35 μm using a cryostat microtome (CM1900, Leica). Free-floating brain sections were first washed with PBS (5 minutes each), blocked with 5% bovine serum albumin (BSA)/0.5% Triton X-100/PBS at a room temperature for 30 minutes, and then incubated with primary antibodies diluted in 3% BSA/0.3% Triton X-100/PBS at 4 °C for 24 hours. After three washes with PBS (5 minutes each), the sections were incubated with secondary antibodies diluted in 0.3% PBST (PBS containing 0.3% Triton X-100) at 37 °C for 1 hour. Finally, the sections were mounted onto glass slides with 50% glycerol-PBS (vol/vol) solution. Images were captured using an Olympus SV120 virtual slide microscope or a confocal laser-scanning microscope (LSM800, Carl Zeiss, Oberkochen, Germany).

### 2.7 RNA-sequencing and bioinformatics analysis

Transcriptome analysis was performed on unilateral hippocampal tissues from 4 mice per group (control group and crotonic acid-treated group). Total RNA was extracted from each model using TRIzol reagent (Invitrogen, USA) following the protocol of manufacturers. The cDNA library construction, library purification, and transcriptome sequencing were conducted in accordance with the instructions provided by Wuhan Huada Sequencing Co., Ltd (a subsidiary of BGI, Shenzhen, China; official website: https://www.genomics.cn/). The volcano plot and heatmap were obtained using the Hiplot website (https://hiplot.com.cn). The differentially expressed gene (DEG) analysis (adjusted *p* < 0.05) was implemented by DESeq2(v1.4.5). Differentially expressed genes (DEGs) were subjected to functional enrichment analysis using Metascape (29) (https://metascape.org/). The analysis included Gene Ontology (GO), Kyoto Encyclopedia of Genes and Genomes (KEGG) pathways. Protein-protein Interaction (PPI) Enrichment Analysis was carried out with the following databases: STRING (30), BioGRid (31), OmniGrid (32), InWeb_IM (33). Gene list enrichments are identified in the following ontology categories: PaGenBase (34), TRRUST (35). The RNA transcriptome sequencing data have been submitted to the Sequence Read Archive database (National Center for Biotechnology Information) (accession number PRJNA1348968).

### 2.8 Gene expression by quantitative real-time PCR

RNA isolation was carried out using the RNAiso Plus reagent (TaKaRa Bio, Kusatsu, Japan) following the protocol of manufacturers. Complementary DNA (cDNA) was synthesized using the PrimeScript™ RT reagent Kit (TaKaRa Bio) as per the instructions of manufacturers. Real-time quantitative PCR (RT-qPCR) was performed with the ChamQ SYBR qPCR Master Mix (Vazyme, Nanjing, China) on a StepOnePlus Real-Time PCR System (Applied Biosystems, Singapore). Relative gene expression was calculated using the 2^(-ΔΔCt) method. Expression values for target genes were normalized to the geometric mean of stable reference genes: ACTB. The relative mRNA expression levels of IL-1β, IL-6 and TNF-α were normalized to the expression of ACTB (β-actin). The primers used were as follows:

ACTB: forward prime, 5’-ATGCCCTGAGGCTCTTTTCC-3’;

reverse primer, 5’-CAGCTCAGTAACAGTCCGCC-3’;

IL-1β: forward prime, 5’-TGCCACCTTTTGACAGTGATG-3’;

reverse primer, 5’-AAGGTCCACGGGAAAGACAC-3’;

IL-6: forward prime, 5’-ACCGCTATGAAGTTCCTCTC-3’;

reverse primer, 5’-CTCTGTGAAGTCTCCTCTCC-3’;

TNF-α: forward prime, 5’-GTGACAAGCCTGTAGCCCAC-3’;

reverse primer, 5’-GCAGCCTTGTCCCTTGAAGA-3’.

### 2.9 Cell culture and treatment

BV2 cell lines were obtained from the Chinese Academy of Sciences Cell Bank (Shanghai, China) and cultured in DMEM/High Glucose medium (Gibco, USA) supplemented with 10% fetal bovine serum (FBS) (Biological Industries, Israel) and 1% penicillin-streptomycin (100 U/Ml penicillin, 100 ug/mL streptomacin, Biological Industries). Cells were seeded at a density of 10,000 cells/well in 96-well plates and treated with fresh complete medium containing crotonic acid at final concentrations of 0 mM (vehicle control, medium only), 0.01 mM, 0.1 mM, and 1.0 mM. Similar as described previously (36), cells were seeded at a density of 10,000 cells/well in 96-well plates and treated with fresh complete medium containing STAT1 inhibitor (Fludarabine, MedChemExpress, USA) at final concentrations of 0 μM (vehicle control, medium only), 0.1 μM, 0.5 μM, 1.0 μM, 5.0 μM and 10.0 μM. For rescue experiments, cells were pretreated with 5 μM fludarabine for 1 h, followed by cotreatment with 0.1mM crotonic acid and 5 μM fludarabine for 6h. Cell viability was assessed using the Cell Counting Kit-8 (CCK-8) (Beyotime, Shanghai, China) following 6 hours of incubation of the cells with crotonic acid at 37 °C in a 5% CO2 humidified incubator.

### 2.10 Subcellular fractionation

BV2 cells were treated with 0 mM and 0.1 mM crotonic acid. Subcellular fractionation was performed using the Nucl-Cyto Preparation Kit (Applygen, Beijing, China) in accordance with the instructions of manufacturers following 6 hours of incubation with crotonic acid. The nuclear fraction was collected and used for Western blotting.

### 2.11 Histone extraction

Histones were extracted from the nuclear fractions of cells or the hippocampal tissues of mice using the Histone Extraction Kit (Abcam, Cambridge, UK) following the protocol of manufacturers. Briefly, samples were lysed with 1× Pre-Lysis Buffer. The mixture was centrifuged at 10,000 rpm (≈5180 x g) for 1 minute at 4 °C and then the pellet was resuspended in Lysis Buffer before incubated on ice for 30 minutes. After centrifugation at 12,000 rpm (≈13800 x g) for 5 minutes at 4 °C, the supernatant was collected, and Balance-DTT Buffer (1 μl DTT Solution to 500 μl Balance Buffer) was added to isolate histones. The extracted histones were subsequently used for immunoblot analysis.

### 2.12 Western blotting

Treated cells and hippocampal tissues were lysed using RIPA lysis buffer and prepared for Western blotting. Proteins were separated on 10% or 12% SDS-polyacrylamide gels by electrophoresis, transferred onto PVDF membranes (Sigma-Aldrich), and blocked for 1 hour with either 5% nonfat milk or 3% BSA, dissolved in TBS-Tween-20 (50 mmol/L Tris-HCl, 150 mmol/L NaCl, 0.2% Tween-20, pH 7.6). Membranes were then incubated with primary antibodies at 4 °C overnight. The following day, membranes were incubated with HRP-conjugated anti-rabbit or anti-mouse secondary antibodies at room temperature for 1 hour, and protein bands were visualized using ECL (Clinx Science Instruments Co., Ltd., China). Immunoblots were quantified using ImageJ (National Institutes of Health, USA). Details of the antibodies used are provided in **Supplementary Table S1**.

### 2.13 Statistical analysis

All data were analyzed and plotted using GraphPad Prism (GraphPad, San Diego, Software). Results are presented as means ± SEM, unless otherwise specified. Statistical analyses were performed using unpaired t-tests or one-way ANOVA, as indicated in the figure legends. A *P*-value < 0.05 was considered statistically significant.

## 3 Results

### 3.1 Histone crotonylation is upregulated in the hippocampus of AD mice

**Figure 1:**
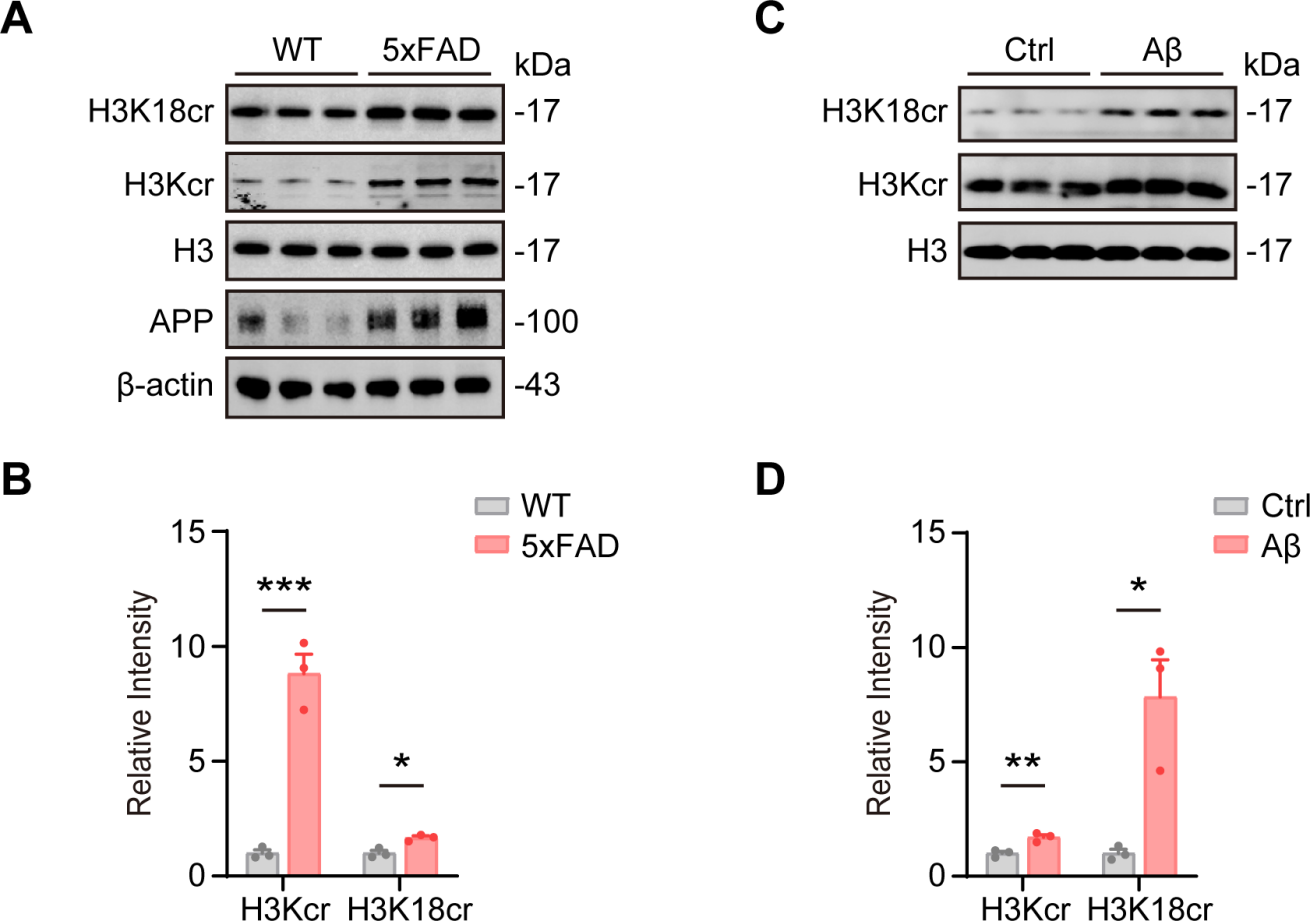
Histone H3 crotonylation is significantly increased in AD mouse models. **(A, B)** Representative immunoblot and corresponding grayscale quantification of pan-histone H3 crotonylation (H3Kcr) and histone H3 lysine 18 crotonylation (H3K18cr) levels in the hippocampus of 8-month-old 5xFAD mice compared to C57BL/6J controls. N = 3 mice per group. Data are presented as mean ± SEM. Two-tailed unpaired t-test, H3Kcr: *t* = 9.085, *P* < 0.001; H3K18cr: *t* = 4.589, *P* < 0.05. **(C, D)** Representative immunoblot and grayscale quantification of H3Kcr and H3K18cr levels in the hippocampus of 2-month C57BL/6J mice two weeks after intracerebroventricular (ICV) injection of Aβ42 compared to controls. N = 3 per group. Data are show as mean ± SEM. Two-tailed unpaired t-test, H3Kcr: *t* = 4.813, *P* < 0.01; H3K18cr: *t* = 4.187, *P* < 0.05.

Previous studies have demonstrated that the 5xFAD mouse model exhibits progressive cognitive impairment, with severity worsening over time (37, 38). Additionally, intracerebroventricular (ICV) injection of Aβ42 in wild-type mice induces cognitive deficits (39). To investigate whether histone crotonylation is altered in AD, we assessed histone crotonylation in these two AD mouse models. Our findings revealed that hippocampal pan-histone H3 crotonylation (H3Kcr) and histone H3 lysine 18 crotonylation (H3K18cr) were significantly upregulated in 8-month-old 5xFAD mice compared to age-matched wild-type controls (**Figures 1A, B**). Similarly, two weeks after ICV injection of Aβ42, hippocampal H3Kcr and H3K18cr were markedly elevated in treated wild-type mice (**Figures 1C, D**). Taken together, these results suggest that histone H3 crotonylation may play a role in AD pathogenesis.

### 3.2 Upregulation of hippocampal histone crotonylation induces cognitive deficits in wild-type mice

**Figure 2:**
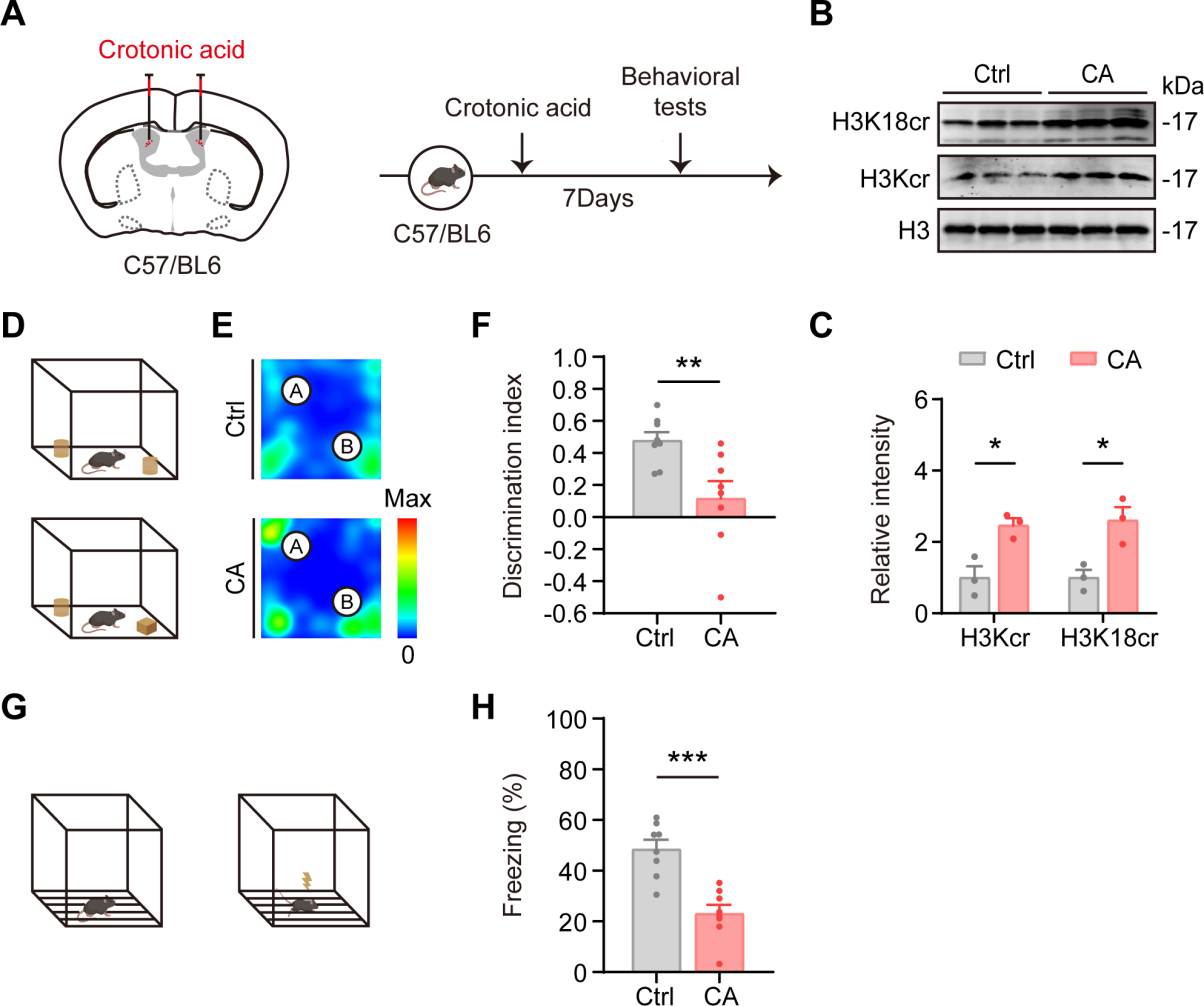
Lateral ventricle injection of crotonic acid increases histone crotonylation and induces cognitive impairment in mice. **(A)** Experimental scheme. C57BL/6J mice were injected with 4 μl of 800mM crotonic acid into the lateral ventricles. Control mice were received 4 μl of 0.9% sterile physiological saline. Behavioral experiments were conducted at 7 days after lateral ventricle injection. **(B, C)** Representative immunoblot and corresponding grayscale quantification of histone crotonylation levels in the control group (Ctrl) and crotonic acid-treated group (CA). N = 3 mice per group. Data are expressed as mean ± SEM. Two-tailed unpaired t-test, H3Kcr: *t* = 3.935, *P* < 0.05; H3K18cr: *t* = 3.744, *P* < 0.05. **(D-F)** Discrimination index of mice in the novel object recognition test. CA mice showed impaired recognition memory compared to Ctrl. N = 8 mice per group. Data are expressed as mean ± SEM. Two-tailed unpaired t-test, *t* = 2.981, *P* < 0.01. **(G, H)** Percentage of freezing time during the contextual fear conditioning test. CA mice exhibited significantly reduced freezing behavior compared to Ctrl, indicating contextual memory impairment. N = 8 mice per group. Data are show as mean ± SEM. Two-tailed unpaired t-test, *t* = 4.954, *P* < 0.001.

To determine whether elevated histone crotonylation contributes to cognitive impairment in AD, we increased hippocampal histone crotonylation levels in mice via ICV injection of crotonic acid. Crotonic acid, a short-chain fatty acid, is converted into crotonyl-CoA *in vivo* and serves as a donor for histone crotonylation. Supplementation with crotonic acid, both *in vivo* and *in vitro*, effectively mimics elevated histone crotonylation (16, 40). Based on *in vitro* validation showing that crotonic acid upregulates H3Kcr and H3K18cr (**Supplementary Figures S1A, B**), we conducted *in vivo* experiments and confirmed that ICV injection of crotonic acid significantly increased H3Kcr and H3K18cr in the hippocampus of wild-type mice (**Figures 2A-C**). Other histone H3 crotonylation sites, as well as pan-histone H3 acetylation, do not exhibit statistically significant differences. (**Supplementary Figures S1H, I**).

Behavioral assessments revealed significant cognitive deficits in the experimental group. There were no significant changes in motor ability in the control group (Ctrl) and crotonic acid-treated group (CA) (**Supplementary Figures S1C, D**). In the novel object recognition test, crotonic acid-treated mice exhibited impaired memory, as evidenced by a marked reduction in exploratory behavior toward the novel object (**Figures 2D-F**). Additionally, in the contextual fear conditioning test, the treated mice showed a significant reduction in freezing behavior compared to controls, indicating impaired contextual memory (**Figures 2G, H**). Anxiety-like behavior was not detected in the open field and elevated plus maze tests (**Supplementary Figures S1E-G**).

Collectively, these findings demonstrate that elevated histone crotonylation in the hippocampus is sufficient to induce cognitive dysfunction in mice, providing novel insights into the potential role of histone crotonylation in AD-related cognitive decline.

### 3.3 Elevated histone crotonylation disrupts synaptic function in wild-type mice

**Figure 3:**
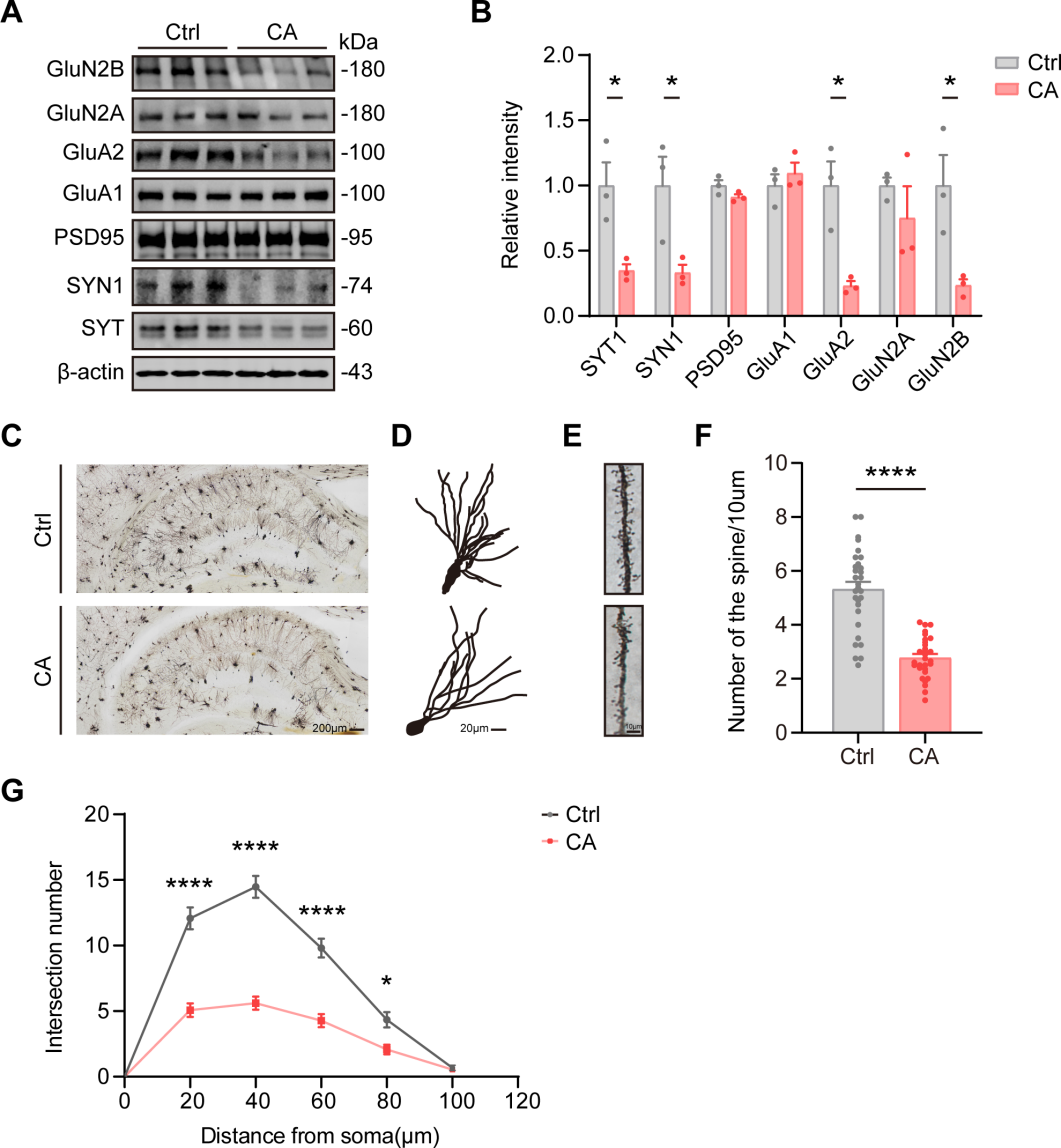
Lateral ventricle injection of crotonic acid reduces synapse-associated proteins and impairs synaptic function. **(A, B)** Representative immunoblot and corresponding grayscale quantification of synapse-associated proteins, including synaptotagmin (SYT), synapsin1 (SYN1), postsynaptic density protein 95 (PSD95), GluA1, GluA2, GluN2A and GluN2B, in the hippocampus of Ctrl and CA mice. N = 3 mice per group. Data are presented as mean ± SEM. Two-tailed unpaired t-test, SYT1: *t* = 3.522, *P* < 0.05; SYN1: *t* = 2.912, *P* < 0.05; PSD95: *t* = 1.903, *P* = 0.1298; GluA1: *t* = 0.7907, *P* = 0.4733. GluA2: *t* = 4.106, *P* < 0.05. GluN2A: *t* = 0.9919, *P* = 0.3774. GluN2B: *t* = 3.214, *P* < 0.05. **(C-F)** Quantification of dendritic spine numbers in the hippocampus of Ctrl and CA mice. Scale bars were indicated in each image as shown. Scale bar, 200 µm (hippocampus), 20 µm (neuron), 10 µm (spine). N = 30 neurons from 3 mice per group. Data are expressed as mean ± SEM. Two-tailed unpaired t-test, *t* = 8.261, *P* < 0.0001. **(G)** Sholl analysis of dendritic complexity in the CA1 region of the hippocampus, showing dendritic crossing in Ctrl and CA mice. N = 15 neurons from three mice for each group. Data are presented as mean ± SEM. Two-way ANOVA, Sidak’s multiple comparisons test, *F*_(5,140)_ = 32.22, *P* < 0.001.

The integrity of neurons and synapses in the brain is critical for maintaining normal cognitive function (41, 42). To investigate whether histone crotonylation impairs cognition by disrupting synaptic function, we first examined the expression of synapse-associated proteins in the hippocampus of mice. Immunoblotting revealed that intraventricular injection of crotonic acid significantly reduced the expression of the presynaptic markers synapsin 1 (SYN1) and synaptotagmin (SYT) in the hippocampus of wild-type mice, while the postsynaptic marker postsynaptic density protein 95 (PSD95) remained unchanged (**Figures 3A, B**). AMPA and NMDA receptors are essential for mediating the release of the excitatory neurotransmitter glutamate from presynaptic vesicles and regulating synaptic plasticity in excitatory neurons (43). Notably, the expression of GluA2 and GluN2B, subunits of AMPA and NMDA receptors, respectively, was significantly reduced in the hippocampus of crotonic acid-treated mice (**Figures 3A, B**).

Dendritic spines, which are the primary sites of synapse formation on neuronal dendrites, play a central role in learning and memory (42). To further examine the effects of histone crotonylation on synaptic structure, we analyzed the morphology and number of dendritic spines in the hippocampus. Golgi staining revealed that the number of dendritic spines in the hippocampus of crotonic acid-treated mice was significantly reduced compared to controls (**Figures 3C-F**). Furthermore, Sholl analysis demonstrated a marked reduction in dendritic complexity in the CA1 region of the hippocampus in the crotonic acid-treated group (**Figure 3G**). These findings indicate that intraventricular injection of crotonic acid induces synaptic damage in the hippocampus, which may underlie the observed cognitive dysfunction.

### 3.4 Upregulation of histone crotonylation triggers microgliosis in wild-type mice

**Figure 4:**
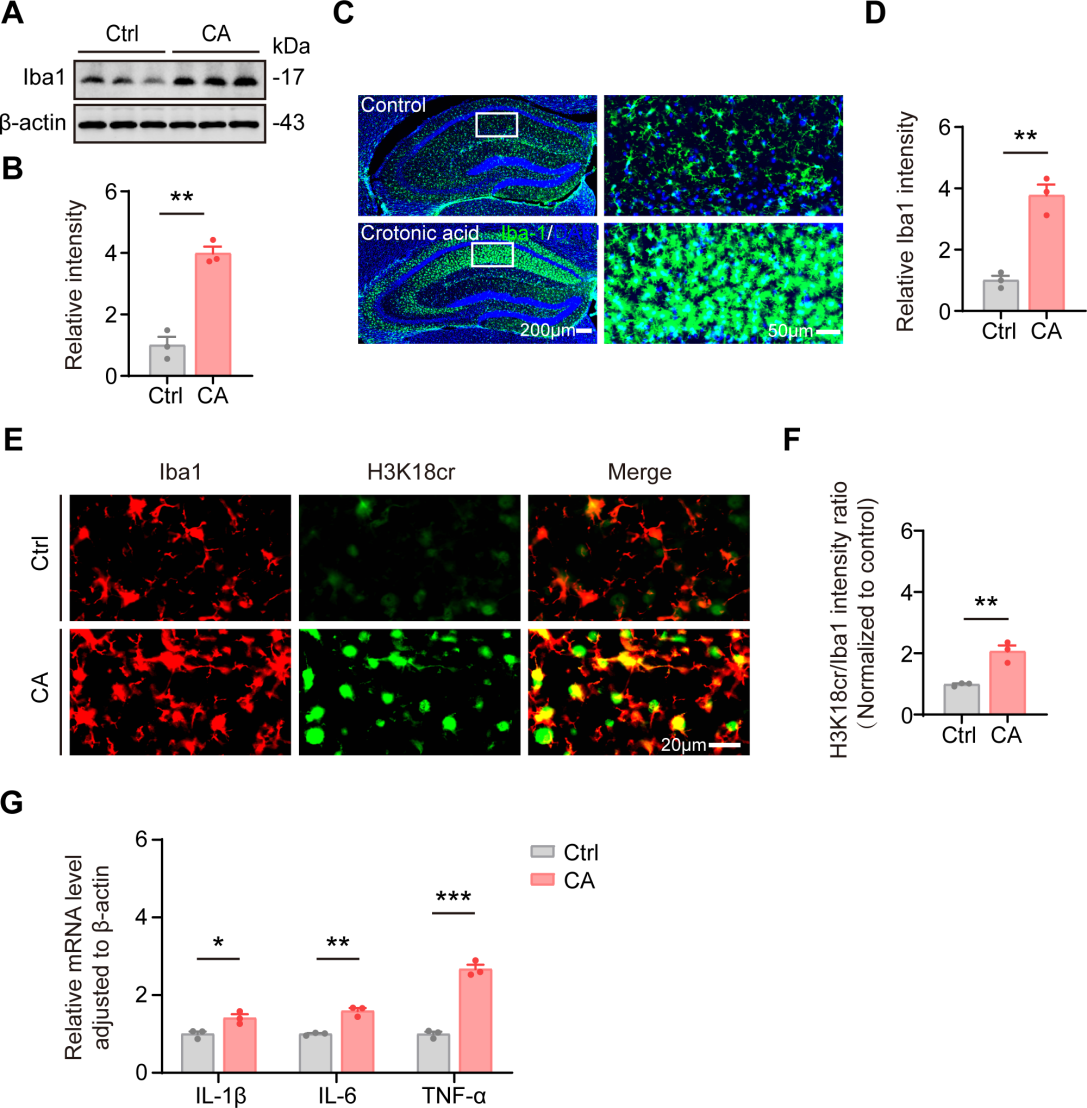
Elevated H3K18cr promotes microglia activation and the release of pro-inflammatory factors. **(A, B)** Representative western blot and corresponding quantitative analysis of Iba1 levels in the hippocampus of mice from the Ctrl and CA mice. N = 3 mice per group. Data are presented as mean ± SEM. Two-tailed unpaired t test, *t* = 8.566, *P* < 0.01. **(C)** Representative immunofluorescence images of Iba1 (microglia marker) staining in the hippocampus of Ctrl and CA. Scale bars, 200 μm or 50 μm. **(D)** Comparison of the relative immunofluorescent intensity of Iba1 in the hippocampal area between Ctrl and CA. N = 3 per group. Data are presented as mean ± SEM. Two-tailed unpaired t test, *t* = 7.314, *P* < 0.01. **(E)** Representative immunofluorescence images showing co-localization of H3K18cr with Iba1 (microglia marker) in the hippocampus of Ctrl and CA. Scale bar, 20 μm. **(F)** Quantification of H3K18cr intensity in Iba1-positive cells. The values were normalized to the control group. N = 3 per group. Data are presented as mean ± SEM. Two-tailed unpaired t test, *t* = 5.405, *P* < 0.01. **(G)** Quantitative of mRNA levels of TNF-α, IL-6 and IL-1β in the hippocampus of Ctrl and CA. N = 3 mice per group. Data are presented as mean ± SEM. Two−tailed unpaired t−test, IL-1β: *t* = 3.669, *P* < 0.05; IL-6: *t* = 7.516, *P* < 0.01; TNF-α: *t* = 13.15, *P* < 0.001.

To investigate the role of histone crotonylation in cognitive impairment in mice, we performed western blot and immunofluorescence analyses to evaluate Iba1 expression in the hippocampus. The results revealed that intraventricular injection of crotonic acid caused a significant upregulation of Iba1 expression in the hippocampus (**Figures 4A, B**), accompanied by pronounced microgliosis (**Figures 4C, D**). Notably, immunofluorescence co-localization showed significantly elevated H3K18cr in hippocampal microglia of the crotonic acid-treated group (**Figures 4E, F**). Immunofluorescence demonstrated no significant difference in the co-localization of H3K18cr with GFAP or NeuN, suggesting that histone crotonylation in neurons and astrocytes was not markedly upregulated following crotonic acid treatment (**Supplementary Figures S2A, B**). To evaluate the potential neurotoxicity of crotonic acid, we conducted NeuN and Nissl staining in the hippocampus. The results demonstrated that neither the number of NeuN-positive neurons (**Supplementary Figure S2C**) nor the morphology of Nissl bodies (**Supplementary Figure S2D**) in the crotonic acid-treated group showed significant differences compared to the control group. These findings suggest that crotonic acid treatment does not induce neuronal loss or structural damage. Additionally, qPCR analysis demonstrated a marked increase in the expression of inflammatory, including IL-1β, IL-6, and TNF-α, in the hippocampus of the crotonic acid-treated group (**Figure 4G**). These findings indicate that intraventricular injection of crotonic acid induces microgliosis and activation, which may contribute to cognitive impairment in mice.

### 3.5 Histone crotonylation significantly activates inflammation-related pathways

**Figure 5:**
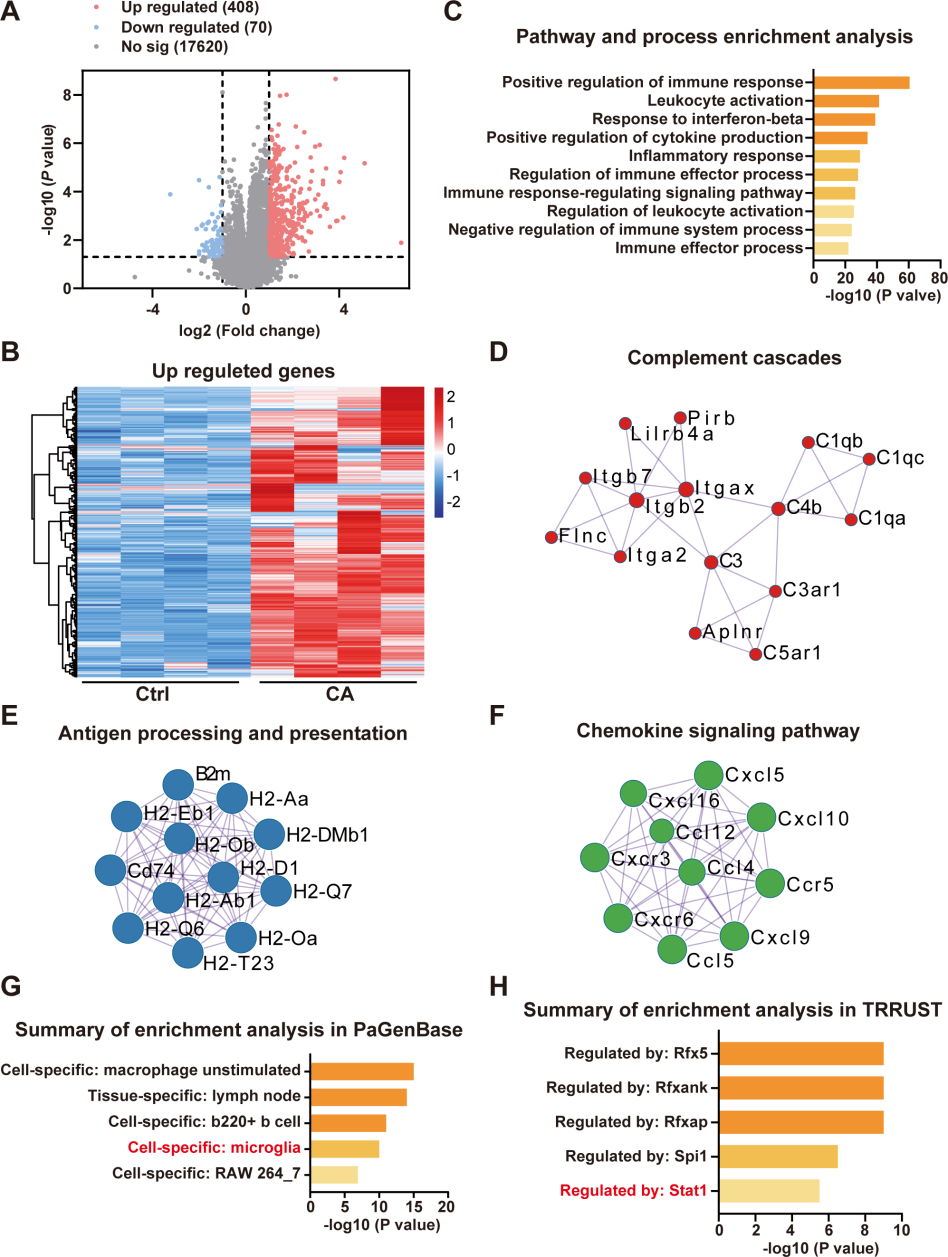
Histone crotonylation significantly activates inflammation-related pathways. **(A)** A total of 408 upregulated and 70 downregulated in 18098 genes were statistically significant in hippocampus in mice after crotonic acid treated measured by RNA-seq analysis. N = 4 mice in each group. **(B)** Heatmap showing gene expression of 408 upregulated differentially expressed genes (DEGs). **(C)** Top Gene Ontology terms for upregulated DEGs. **(D-F)** Protein-protein interaction network (PPI) analysis shown the key molecule network in upregulated DEGs. **(G)** Summary of enrichment analysis in PaGenBase module of Metascape across upregulated genes in CA. **(H)** Comparison of transcription regulators with enriched targets among upregulated genes, as identified by the TRRUST module of Metascape.

To further investigate the molecular mechanisms underlying microgliosis, we performed transcriptomic sequencing on hippocampal tissues from wild-type mice following crotonic acid injection into the lateral ventricle. This analysis identified 478 differentially expressed genes (DEGs), of which 408 were upregulated and 70 were downregulated (**Figures 5A, B**). To comprehensively characterize the roles of the upregulated genes, we conducted functional analysis using the metascape bioinformatics database. The results revealed that these upregulated DEGs were predominantly enriched in immune-related signaling pathways (**Figure 5C**). Protein-protein interaction analysis further highlighted significant enrichment in several inflammation-related pathways, including complement cascades, antigen processing and presentation, and chemokine signaling pathway (**Figures 5D-F**). Enrichment analysis using PaGenBase demonstrated that these genes were almost exclusively expressed in microglia (**Figure 5G**). Moreover, upstream regulator analysis demonstrates strong enrichment of signal transducer and activator of transcription 1 (STAT1) targets among upregulated genes following crotonic acid treatment (**Figure 5H**, **Supplementary Figure S3**). These findings suggest that histone crotonylation, through the promotion of STAT1 activation and microglia-mediated neuroinflammation, may play a critical role in memory impairment.

### 3.6 An enhanced H3K18cr upregulates microglia expression of pro-inflammation-related transcription factor STAT1

**Figure 6:**
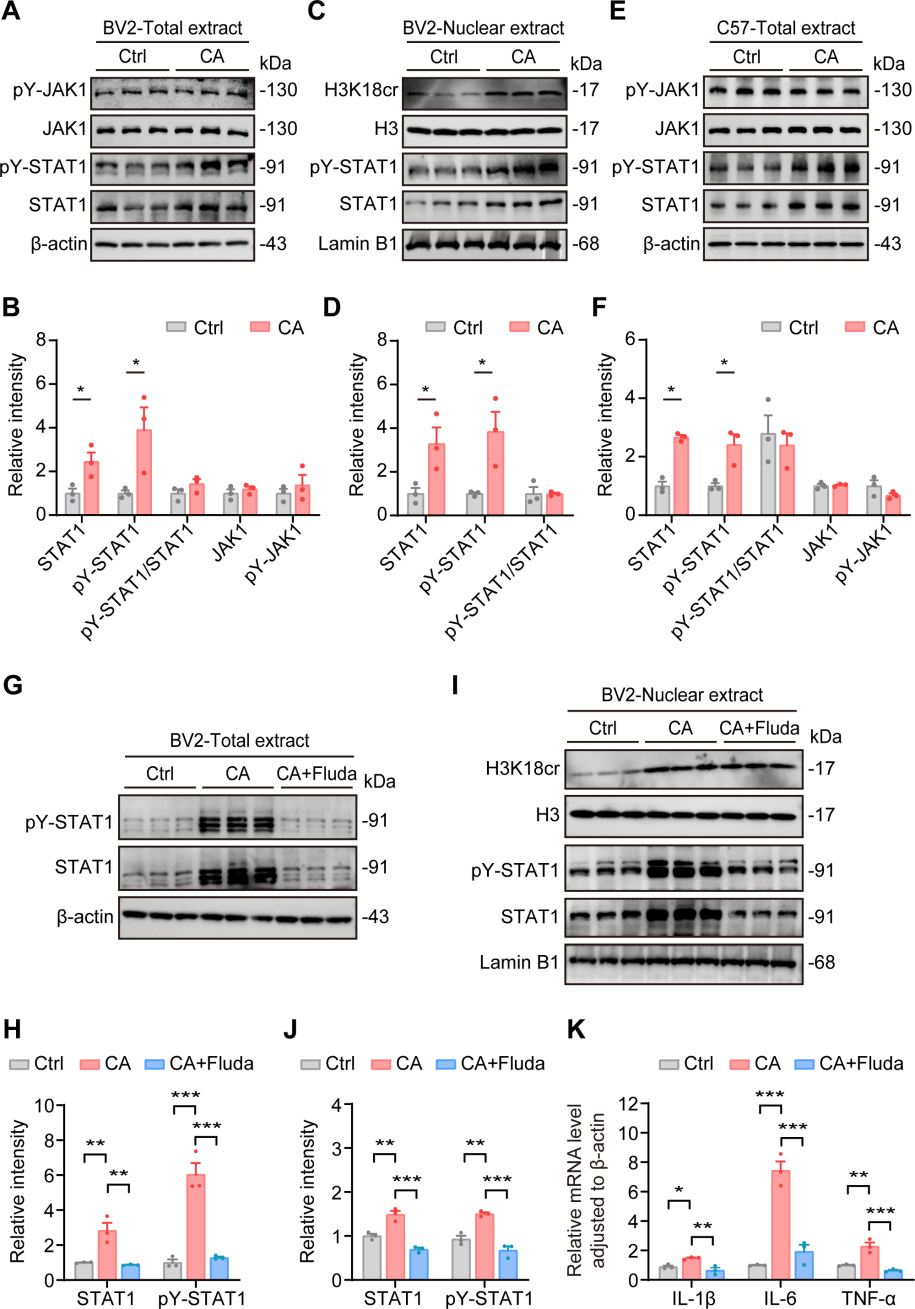
An enhanced H3K18cr upregulates microglia expression of pro-inflammation-related transcription factor STAT1. **(A, B)** Representative western blot images and corresponding quantitative analysis of STAT1, pY-STAT1, JAK1 and pY-JAK1 levels in BV2 cells treated with 0.1 mM crotonic acid for 6 hours. N = 3 per group. Data are presented as mean ± SEM. Two-tailed unpaired t-test, STAT1: *t* = 3.084, *P* < 0.05; pY-STAT1: *t* = 2.802, *P* < 0.05; pY-STAT1/STAT1: *t* = 1.553, *P* = 0.1955; JAK1: *t* = 0.8888, *P* = 0.4243. pY-JAK1: *t* = 0.7728, *P* = 0.4828. **(C, D)** Representative western blot images and quantitative analysis of H3, H3K18cr, STAT1 and pY-STAT1 levels in BV2 nuclear extracts following 0.1 mM crotonic acid treatment for 6 hours. N = 3 per group. Data are presented as mean ± SEM. Two-tailed unpaired t-test, STAT1: *t* = 2.934, *P* < 0.05; pY-STAT1: *t* = 3.168, *P* < 0.05; pY-STAT1/STAT1: *t* = 0.01308, *P* = 0.9902. **(E, F)** Representative western blot images and corresponding quantitative analysis of STAT1, pY-STAT1, JAK1 and pY-JAK1 levels in the hippocampus of mice from the Ctrl and CA. N = 3 per group. Data are presented as mean ± SEM. Two-tailed unpaired t-test, STAT1: *t* = 9.929, *P* < 0.001; pY-STAT1: *t* = 3.814, *P* < 0.05; pY-STAT1/STAT1: *t* = 0.5361, *P* = 0.6203; JAK1: *t* = 0.3434, *P* = 0.7486. pY-JAK1: *t* = 1.583, *P* = 0.1887. **(G, H)** Representative western blot images and corresponding quantitative analysis of STAT1 and pY-STAT1 levels in BV2 cells treated with 0.1 mM crotonic acid and 5.0 μM fludarabine. N = 3 per group. Data are presented as mean ± SEM. One-way ANOVA test followed by Tukey’s multiple comparisons test, STAT1: interaction *F*_(2,6)_ = 19.02, *P* < 0.01; pY-STAT1: interaction *F*_(2,6)_ = 50.90, *P* < 0.001. **(I, J)** Representative western blot images and corresponding quantitative analysis of STAT1 and pY-STAT1 levels in BV2 nuclear extracts following 0.1 mM crotonic acid and 5.0 μM fludarabine. N = 3 per group. Data are presented as mean ± SEM. One-way ANOVA test followed by Tukey’s multiple comparisons test, STAT1: interaction *F*_(2,6)_ = 45.52, *P* < 0.001; pY-STAT1: interaction *F*_(2,6)_ = 35.12, *P* < 0.001. **(K)** Quantitative of mRNA levels of TNF-α, IL-6 and IL-1β in BV2 cells. N = 3 mice per group. Data are presented as mean ± SEM. One-way ANOVA test followed by Tukey’s multiple comparisons test, IL-1β: interaction *F*_(2,6)_ = 12.21, *P* < 0.01; IL-6: interaction *F*_(2,6)_ = 59.86, *P* < 0.001; TNF-α: interaction *F*_(2,6)_ = 30.37, *P* < 0.001.

To elucidate the molecular mechanism by which crotonic acid upregulates H3K18cr and activates microglia, we hypothesized, based on transcriptomic data, that STAT1 is a critical factor driving microglia activation. Previous studies have shown that STAT1 regulates the expression of pro-inflammatory factors in microglia (44). We initially validated the crotonylation-inducing effect of crotonic acid in N2a neuroblastoma cells (**Supplementary Figures S1A, B**), then proceeded to investigate microglial-specific responses using BV2 cells. To test this hypothesis, we quantified the expression levels of transcription factors in BV2 cells treated with 0.1 mM crotonic acid for 6 hours. Western blot analysis revealed a significantly upregulation of STAT1 protein levels, accompanied by a proportional increase in the phosphorylation of its key activation site, Tyr701 (pY-STAT1) (**Figures 6A, B**). However, no significant changes were observed in the phosphorylation levels of STAT1’s upstream tyrosine kinase, Janus kinase 1 (JAK1), at its Tyr1034/1035 site (pY-JAK1) (**Figures 6A, B**). Typically, JAK1 undergoes autophosphorylation upon receiving extracellular signals and subsequently catalyzes STAT1 phosphorylation at Tyr701. The phosphorylated STAT1 (pY-STAT1) then forms dimers and translocates to the nucleus, where it regulates the transcription of pro-inflammatory factors, including TNF-α, IL-6, and IL-1β. Our findings indicate that crotonic acid does not activate the STAT1 pathway via JAK1. Consistently, crotonic acid treatment significantly increased nuclear levels of STAT1 and pY-STAT1 in BV2 cells, with no significant change in their relative ratio (**Figures 6C, D**). Treatment with the STAT1 inhibitor fludarabine significantly restores the total and nuclear levels of STAT1 and pY-STAT1 in BV2 cells following crotonic acid exposure (**Figures 6G–J**). Additionally, fludarabine treatment markedly rescues the mRNA levels of pro-inflammatory cytokines, including TNF-α, IL-6, and IL-1β, in BV2 cells after crotonic acid treatment (**Figure 6K**).

These results suggest that crotonic acid upregulates STAT1 expression in BV2 cells, which is consistent with the observed increase in total STAT1 levels in the hippocampus following intraventricular crotonic acid injection *in vivo* (**Figures 6E, F**). Furthermore, crotonic acid treatment did not alter the expression of other transcription factors, such as STAT3, pY-STAT3 or NF-κB in BV2 cells or in the hippocampus of C57 mice (**Supplementary Figure S4**). In summary, these findings suggest that crotonic acid enhances the release of pro-inflammatory factors in microglia by upregulating H3K18cr and stimulating the transcription of the transcription factor STAT1.

## 4 Discussion

This study systematically elucidates the critical role of histone crotonylation in the pathogenesis of AD, offering novel insights into the epigenetic mechanisms underlying this devastating disorder. For the first time, we observed altered histone crotonylation levels in hippocampal tissues from two well established AD mouse models, 5xFAD transgenic mice and Aβ42 lateral ventricle injection mice, thereby linking this epigenetic modification to AD pathology. Notably, we established a causal relationship between histone crotonylation and cognitive dysfunction by injecting crotonic acid into the lateral ventricles of wild-type mice, which induced AD-like cognitive impairments. This discovery adds new information to the existing paradigms of AD epigenetic mechanisms and extends our understanding of its molecular underpinnings.

Importantly, we identified a significant elevation of H3K18cr levels in microglia and uncovered the histone crotonylation/STAT1 signaling axis as a previously unrecognized molecular pathway driving microglial overactivation and neuroinflammation. Crotonic acid administration markedly increased STAT1 expression in microglia, which subsequently promoted the production of pro-inflammatory cytokines, including IL-1β, IL-6, and TNF-α. Collectively, these findings provide the first comprehensive evidence chain linking metabolite-driven histone modifications to AD pathogenesis via microglial dysfunction, offering a novel perspective on the disease’s etiology. Building on these insights, it is important to explore how the H3K18cr-STAT1 axis integrates with classical AD pathologies, such as Aβ accumulation and tau hyperphosphorylation, to further elucidate its role in disease progression. The H3K18cr-STAT1 axis likely interacts bidirectionally with classical AD pathologies. Aβ oligomers can trigger metabolic dysfunction in microglia, potentially increasing crotonyl-CoA production and subsequent H3K18cr levels (45). Conversely, STAT1-driven neuroinflammation may impair Aβ clearance mechanisms and promote tau hyperphosphorylation through inflammatory kinase activation. Recent evidence suggests that pro-inflammatory cytokines like IL-1β and TNF-α, which are upregulated by STAT1, can enhance β-secretase activity and tau phosphorylation (46, 47). Furthermore, chronic microglial activation may compromise the blood-brain barrier, facilitating Aβ accumulation (48). Future studies should investigate whether targeting the H3K18cr-STAT1 axis can modulate Aβ and tau pathology, potentially offering a novel therapeutic approach that addresses both neuroinflammation and proteinopathy in AD.

AD is a multifactorial disorder with a complex etiology involving both genetic and environmental factors. Environmental influences can induce epigenetic modifications of AD-related genes (49). Among these, post-translational modifications of histones are emerging as key contributors to AD pathogenesis (50). Compared to traditional histone acetylation and methylation, histone crotonylation exhibits unique biochemical properties, including greater hydrophobicity and steric hindrance, which likely exert more pronounced effects on chromatin structure and gene transcription (11, 12). Evidence is mounting that histone crotonylation plays a role in various physiological and pathological processes, including differentiation (11, 51), tissue injury (52), viral infection (15, 53), tumorigenesis (54), and neurodegenerative diseases (55, 56). The abnormal elevation of H3K18cr levels in hippocampal tissues may represent a novel pathological hallmark of AD, potentially arising from increased crotonyl-CoA levels in the brain. Crotonyl-CoA is primarily derived from crotonic acid through the Acyl-CoA Synthetase Short Chain Family Member 2 (ACSS2) (16). Investigating whether crotonic acid levels are elevated in AD brains and identifying their sources warrant further study. Potential mechanisms include altered metabolic pathways, such as impaired fatty acid β-oxidation, mitochondrial dysfunction leading to metabolic reprogramming, dysregulated short-chain fatty acid metabolism due to gut microbiota imbalance, and increased blood-brain barrier permeability facilitating exogenous crotonic acid accumulation (57, 58). The specificity of H3K18cr as a biomarker lies in its association with cognitive dysfunction, while its reversibility provides a theoretical basis for therapeutic intervention. Modulating the balance between crotonylation and decrotonylation enzymes may offer a novel strategy to reverse pathological changes.

Our findings demonstrate that crotonylation-induced synaptic damage exhibits selective vulnerability. Specifically, presynaptic markers such as SYN1 and SYT were significantly reduced, whereas PSD95 was relatively preserved, suggesting that presynaptic functions are more sensitive to histone crotonylation. This selective vulnerability may stem from the dynamic nature of the presynaptic membrane and its susceptibility to metabolic disturbances. Additionally, the observed reductions in AMPA receptor subunit GluA2 and NMDA receptor subunit GluN2B impair synaptic transmission efficiency and plasticity. The loss of GluA2 alters calcium permeability of AMPA receptors, while reduced GluN2B levels compromise the induction and maintenance of long-term potentiation (LTP). Together, these changes contribute to hippocampus-dependent deficits in learning and memory. We observed a reduction in dendritic spine density and morphological complexity, aligning closely with synaptic pathology characteristic of classical AD but occurring at earlier stages. This suggests that histone crotonylation may act as an upstream event in synaptic damage. Cognitive deficits observed in behavioral tests, such as impaired novel object recognition and weakened contextual fear memory, correlate with the extent of synaptic damage. These findings, spanning molecular to behavioral levels, underscore the central role of histone crotonylation in regulating cognitive functions.

Microglial activation emerges as a central mediator of crotonylation-induced pathological changes. A significant upregulation of Iba1 expression, accompanied by a morphological shift from ramified to amoeboid microglia, indicates their transition from a resting to an activated state (59). This activation pattern shares similarities with disease-associated microglia (DAM) phenotypes observed in classical AD pathology while exhibiting unique characteristics. Analysis of inflammatory cytokine profiles revealed a synergistic elevation of IL-1β, IL-6, and TNF-α, creating a robust pro-inflammatory microenvironment. Compared to traditional AD-associated inflammation, crotonylation-induced inflammation initiates more rapidly and progresses more intensely. Notably, significant colocalization of H3K18cr in microglia provides direct evidence of cell type-specific epigenetic reprogramming. Studies on histone lactylation have shown its influence on microglial functional states via metabolic reprogramming (60, 61). Similarly, elevated histone crotonylation in medullary microglia in neuropathic pain models induces microglial activation and pro-inflammatory factor expression, while reducing histone crotonylation levels ameliorates these phenotypes (26). Another study demonstrated that H3K27cr promotes microglial phagocytosis of Aβ by regulating endocytosis-related gene expression (56). Our findings align with these observations, suggesting that histone crotonylation in microglia alters chromatin accessibility of inflammation-related genes, thereby promoting pro-inflammatory transcriptional programs. A potential positive feedback loop may exist wherein crotonylation enhances inflammatory factor release, which further alters cellular metabolism to produce more crotonic acid. This exacerbates histone crotonylation, perpetuating a vicious cycle. This mechanism may explain the persistent and progressive nature of neuroinflammation in AD, though further experimental validation is required.

Transcriptomic analysis revealed strong enrichment of immune-related pathways, with significant upregulation of STAT1 target genes. TRUSST analysis identified STAT1 as a key transcription factor downstream of crotonylation. STAT1 plays a pivotal role in immune responses and inflammation (62). The transcriptomic changes observed could partially reflect increased microglial density rather than purely transcriptional reprogramming. Future studies should employ single-cell RNA sequencing or isolated microglial populations to definitively distinguish between these possibilities. Elevated STAT1 expression and phosphorylation have been observed in AD brains, correlating positively with cognitive decline (63). Activated STAT1 promotes the expression of various pro-inflammatory factors and chemokines, exacerbating neuroinflammatory damage (44, 64). Interestingly, in this study, STAT1 activation occurred independently of classical JAK1 phosphorylation, suggesting a non-canonical activation mechanism. This process may involve the enrichment of crotonylated histones at the promoter region of STAT1 gene. Compared to other microglia activation-related transcription factors, such as NF-κB and STAT3, the STAT1-mediated signaling pathway played a dominant role in histone crotonylation induced inflammatory responses. The selective upregulation of STAT1 likely results from direct transcriptional regulation by histone crotonylation. The enrichment of H3K18cr at transcription initiation sites further supports its role as an upstream regulator of STAT1 expression and activation. A key limitation of our study is the lack of ChIP-seq data demonstrating direct enrichment of H3K18cr at the STAT1 promoter or enhancer regions. While our data strongly suggest that H3K18cr upregulates STAT1 transcription, establishing this direct epigenetic regulation requires chromatin immunoprecipitation experiments, which represents an important future direction for this work. We believe our current evidence, including transcriptomic data showing STAT1 as the top enriched transcription factor and the temporal correlation between H3K18cr and STAT1 upregulation, provides strong correlative support for our hypothesis.

Our acute crotonic acid administration model, while effective in inducing AD-like changes, does not capture the chronic, progressive nature of AD. The 7-day treatment period may induce compensatory mechanisms that differ from those in chronic disease. Long-term, low-dose crotonic acid exposure, perhaps over one or more months, would better model the gradual accumulation of epigenetic changes in AD. Additionally, examining histone crotonylation at different disease stages in aging 5xFAD mice would reveal the temporal dynamics of this modification in AD progression. A crucial next step is validating our findings in human AD brain tissues. Future studies should examine H3K18cr levels in postmortem AD brain samples, particularly in isolated microglia, and correlate these with disease severity and STAT1 expression. Additionally, investigating H3K18cr in patient-derived induced pluripotent stem cell models would provide a human-relevant system for mechanistic studies and drug screening.

While the acute crotonic acid administration model successfully induced AD-like pathological changes, it does not fully replicate the chronic progression of the disease. Long-term, low-dose exposure may better simulate the natural course of AD and reveal dynamic changes in histone crotonylation at different disease stages. Validation in human AD brain tissues and patient-derived cells is essential to confirm the clinical relevance of these findings. Furthermore, AD affects multiple brain regions, including the cortex, amygdala, and basal forebrain, which may exhibit distinct histone crotonylation patterns. Comprehensive studies across these regions are warranted. The lack of temporal analyses limits our understanding of whether histone crotonylation serves as an early driver or a late consequence in the AD pathological cascade. Additionally, systemic modulation of histone crotonylation must be evaluated for potential off-target effects, given its role in various physiological processes. Our exclusive use of male mice is a significant limitation, particularly given the female predominance in AD prevalence and potential sex differences in microglial responses. This decision was made to reduce variability in this initial study, but future work must include both sexes to ensure translational relevance. Finally, the interaction between histone crotonylation and classical AD pathologies, such as Aβ plaques and tau tangles, remains unexplored and may hold critical implications for understanding disease mechanisms and developing therapeutic strategies.

## 5 Conclusions

This study identifies H3K18cr as a novel epigenetic mechanism driving the pathogenesis of AD, providing significant insights into the molecular basis of this complex disorder. By demonstrating how crotonic acid-induced H3K18cr lead to neuroinflammation and cognitive impairment via STAT1-dependent microglial activation, we propose a new paradigm in which metabolite-driven epigenetic changes actively contribute to neurodegenerative diseases. These findings not only deepen our understanding of AD pathophysiology but also highlight H3K18cr as a potential therapeutic target.

## Data Availability

The datasets presented in this study can be found in online repositories. The names of the repository/repositories and accession number(s) can be found below: PRJNA1348968 (SRA).
