# Supplementary material for: Microglial histone H3K18 crotonylation promotes STAT1 expression and induces cognitive deficit in Alzheimer disease
Source: Front Immunol. 2026 Jan 27;17:1744375. doi: 10.3389/fimmu.2026.1744375 (PMC12886003; doi:10.3389/fimmu.2026.1744375)
Supplement: Supplementary file 1 [file DataSheet1.pdf]

Supplementary Material

Supplementary Figures and Tables

Supplementary Figures

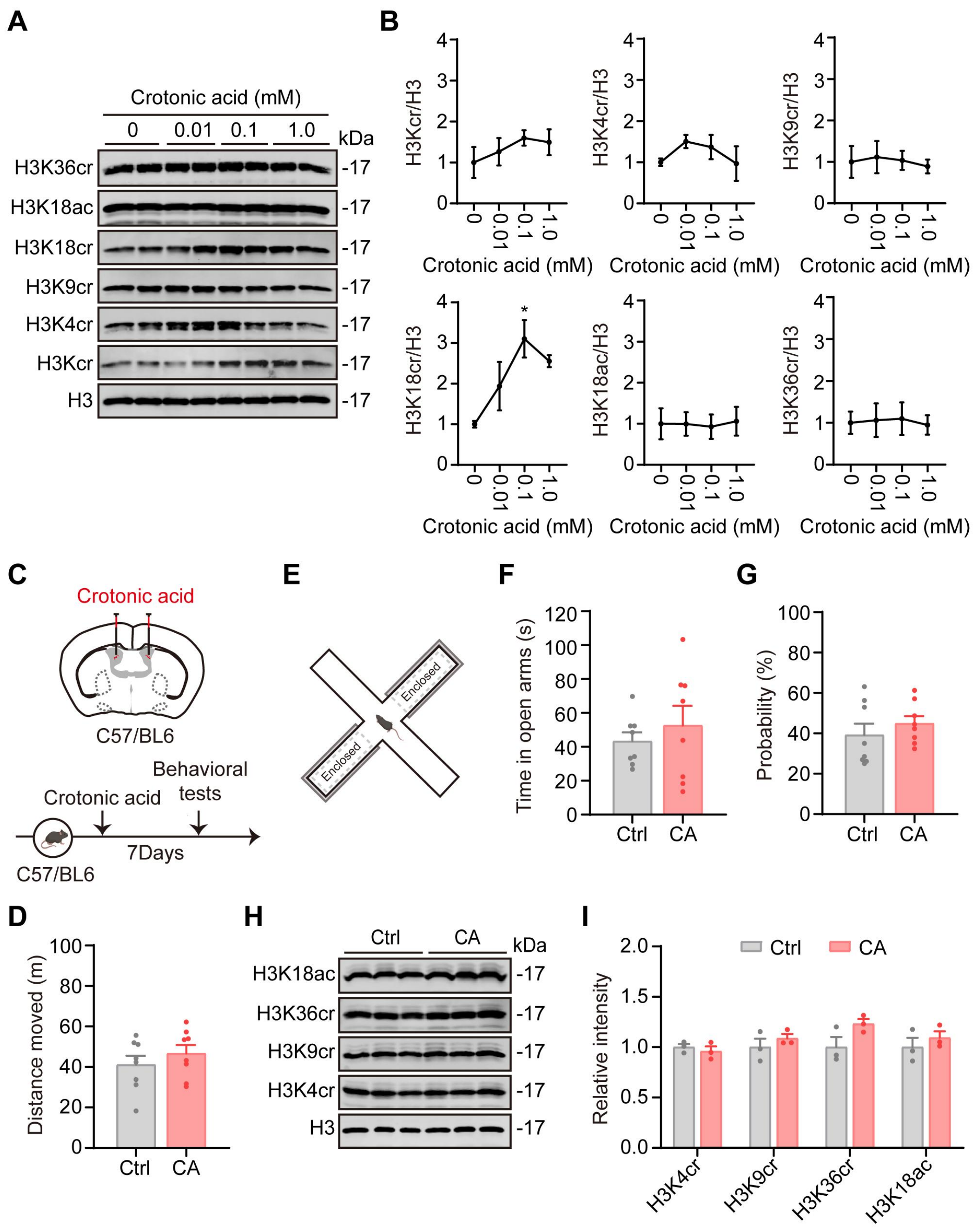

Supplementary Figure 1. Crotonic acid upregulates histone crotonylation in vitro. (A, B). Crotonic acid induces an increased histone H3 lysine crotonylation at 0.01 and 0. 1 mM in N2a cells measured by Western blotting. Data are presented as mean ± SEM. One-way ANOVA test followed by Tukey's multiple comparisons test, H3Kcr/H3: interaction $F_{(3,8)}$ = 0.7240, P = 0.5655; H3K4cr/H3: interaction $F_{(3,8)}$ = 0.9611, P = 0.4566; H3K9cr/H3: interaction $F_{(3,8)}$ = 0.09266, P = 0.9620; H3K18cr/H3: interaction $F_{(3,8)}$ = 5.451, P < 0.05; H3K18ac/H3: interaction $F_{(3,8)}$ = 0.02706, P = 0.9935; H3K36cr/H3: interaction $F_{(3,8)}$ = 0. 04021, P = 0.9884. **(C).** Experimental scheme. **(D).** There was no meaningful difference for distance moved in the control group (Ctrl) and crotonic acid-treated group (CA). N = 8 mice per group. Data are expressed as mean ± SEM. Two-tailed unpaired t test, t = 0.9142, P = 0.3761. **(E-G).** The elevated plus maze revealed no significant difference for anxiety in the Ctrl and CA mice. N = 8 mice per group. Data are expressed as mean ± SEM. Two-tailed unpaired t test, t = 0.7249, P = 0.4804 (F); t = 0.8549, P = 0.4070 (D).Unpaired T test. **(H, I).** Representative immunoblot and corresponding grayscale quantification of histone crotonylation levels in the Ctrl and CA. N = 3 mice per group. Data are expressed as mean ± SEM. Two-tailed unpaired t-test. H3K4cr: t = 0.7139, P = 0.5148; H3K9cr: t = 0.9117, P = 0.4135; H3K36cr: t = 2.064, P = 0.1080; H3K18ac: t = 0.8299, P = 0.4533.

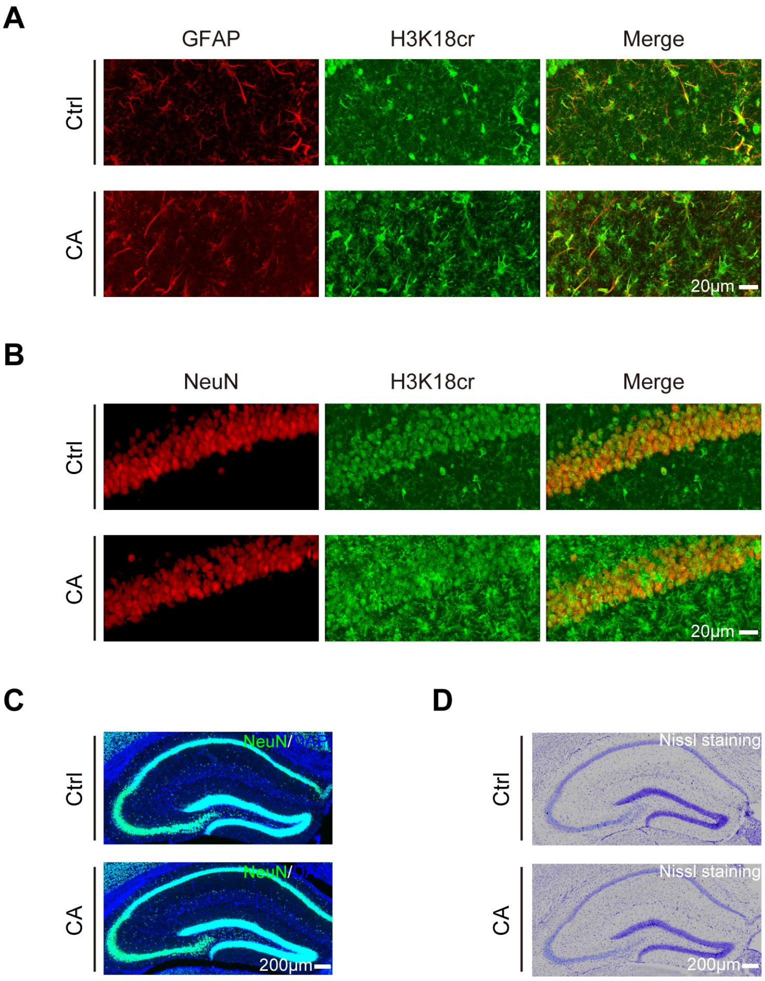

Supplementary Figure 2. No obvious upregulation of histone crotonylation in neurons and astrocytes were observed after intracerebroventricular injection of crotonic acid. (A). Representative immunofluorescence images showing co-localization of H3K18cr with GFAP (astrocytes marker) in the hippocampus of Ctrl and CA. Scale bar, 20 μm. **(B).** Representative immunofluorescence images showing co-localization of H3K18cr with NeuN (Neurons marker) in the hippocampus of Ctrl and CA. Scale bar, 20 μm. **(C).** Representative immunofluorescence images of NeuN in the hippocampus of Ctrl and CA. Scale bar, 200 μm. **(D).** Representative nissl staining in the hippocampus of Ctrl and CA. Scale bar, 200 μm.

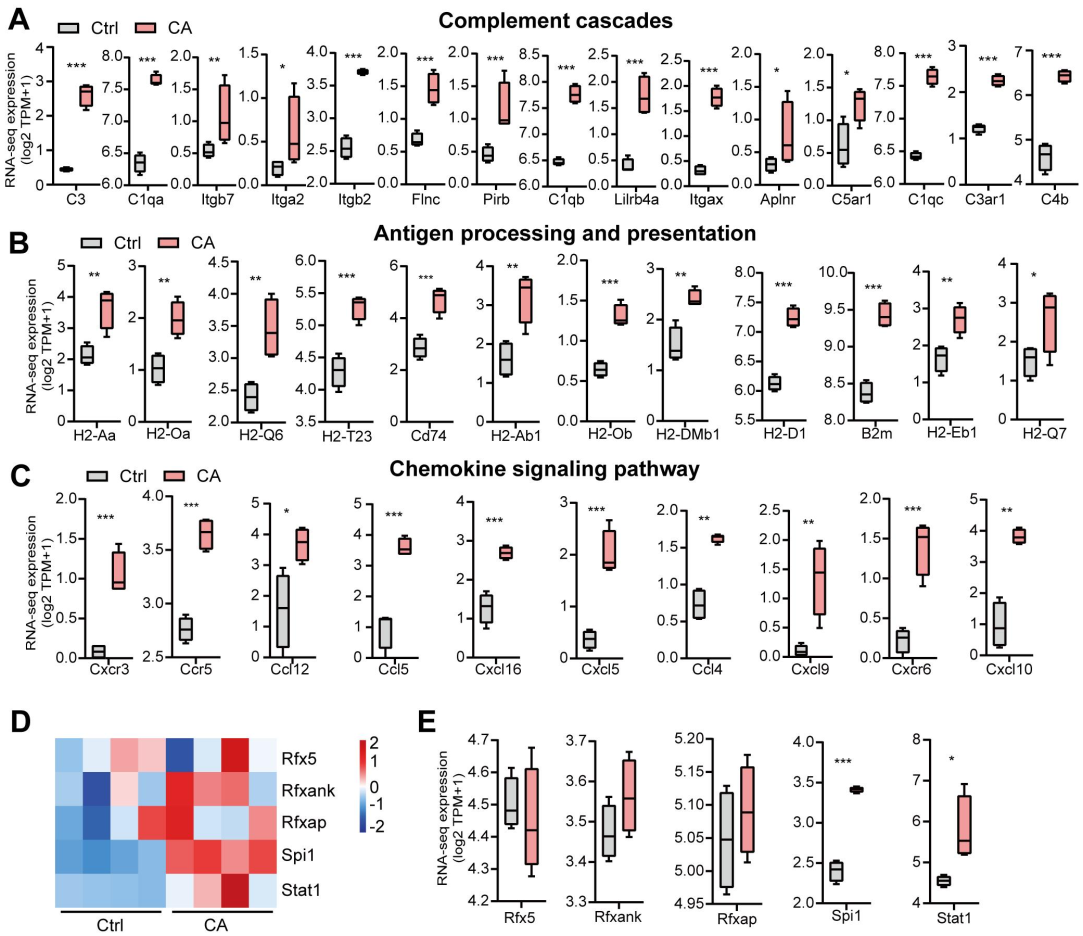

Supplementary Figure 3. Gene expression of PPI analysis and top five associated transcription factors identified by the TRRUST module of Metascape. (A-C). Gene expression of key molecule network of PPI analysis in upregulated DEGs. RNA-seq expression (log2 TPM + 1) was visualised as boxplots. **(D)** Heatmap illustrating the gene expression profiles of the top five transcription factors identified by the TRRUST module of Metascape. **(E).** Top five associated transcription factors expression in crotonic acid-treated group targets upregulated DEGs, as identified by the TRRUST module of Metascape. RNA-seq expression (log2 TPM + 1) was visualised as boxplots. N = 4 per group.

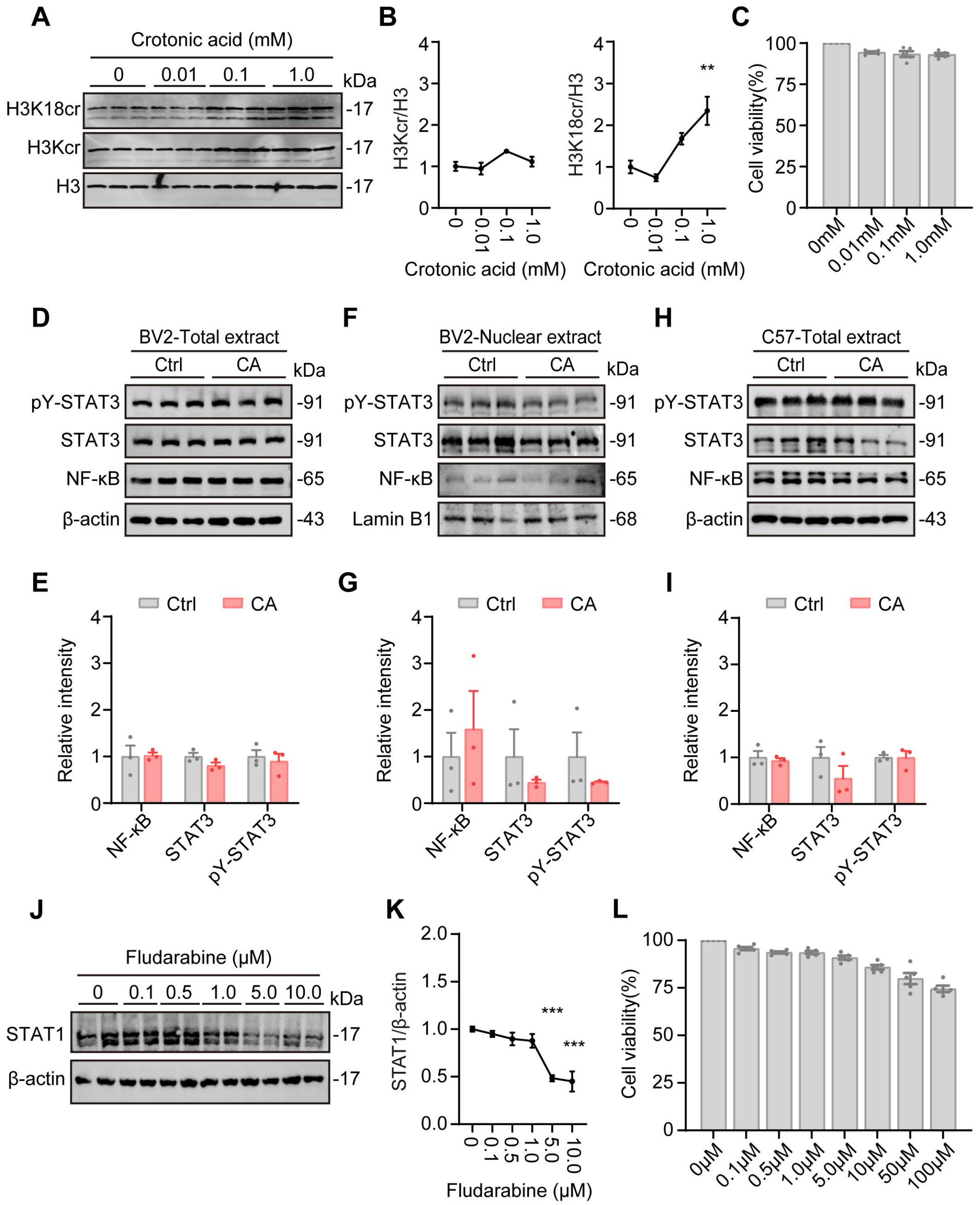

Supplementary Figure 4. Crotonic acid treatment did not alter the expression of other transcription factors. (A, B). Crotonic acid didn't induce a concentration-dependent clearance of histone crotonylation in BV2 cells measured by Western blotting. Data are presented as mean ± SEM. One-way ANOVA test followed by Tukey's multiple comparisons test, H3Kcr/H3: interaction $F_{(3,8)}$ = 2.962, P = 0.0975; H3K18cr/H3: interaction $F_{(3,8)}$ = 12.73, P < 0.01. **(C).** Compared with the vehicle, Crotonic acid (0.01-1.0 mM for 6 h) did not significantly affect cell viability measured by CCK-8 assay in BV2 cells. **(D, E).** Representative western blot images and corresponding quantitative analysis of STAT3, pY-STAT3 and NF-κB levels in

BV2 cells treated with 0.1 mM crotonic acid for 6 hours. N = 3 per group. Data are presented as mean ± SEM. Two-tailed unpaired t-test, NF-κB: $t = 0.09018$, $P = 0.9325$; STAT3: $t = 1.911$, $P = 0.1286$; pY-STAT3: $t = 0.4824$, $P = 0.6547$. **(F, G).** Representative western blot images and quantitative analysis of STAT3, pY-STAT3 and NF-κB levels in BV2 nuclear extracts following 0.1 mM crotonic acid treatment for 6 hours. N = 3 per group. Data are presented as mean ± SEM. Two-tailed unpaired t-test, NF-κB: $t = 0.6125$, $P = 0.5733$; STAT3: $t = 0.9377$, $P = 0.4015$; pY-STAT3: $t = 1.058$, $P = 0.3499$. **(H, I).** Representative western blot images and corresponding quantitalive analysis of STAT3, pY-STAT3 and NF-κB levels in the hippocampus of mice from the Ctrl and CA. N = 3 per group. Data are presented as mean ± SEM. Two-tailed unpaired t-test, NF-κB: $t = 0.4666$, $P = 0.6651$; STAT3: $t = 1.284$, $P = 0.2686$; pY-STAT3: $t = 0.01992$, $P = 0.9851$. **(J, K).** Fludarabine didn't induce a concentration-dependent clearance of STAT1 decrease in BV2 cells measured by Western blotting. Data are presented as mean ± SEM. One-way ANOVA test followed by Tukey's multiple comparisons test, interaction $F_{(5,12)} = 15.21$, P j< 0.0001. **(L).** Compared with the vehicle, Fludarabine (0.1-100 μM for 6 h) affected cell viability measured by CCK-8 assay in BV2 cells.

Supplementary Table1: Details of the antibodies

Antibody	Source	Catalogue	
H3	PTM BIO	PTM-6613	1:1000(WB)
H3K4cr	PTM BIO	PTM-527	1:1000(WB)
H3K9cr	PTM BIO	PTM-539	1:1000(WB)
H3K18cr	PTM BIO	PTM-540	1:1000(WB)
H3K18ac	PTM BIO	PTM-114RM	1:1000(WB)<br>1:300(IF)
H3K36cr	PTM BIO	PTM-536RM	1:1000(WB)
H3Kcr	PTM BIO	PTM-501	1:1000(WB)
APP	Cell Signaling	29765	1:1000(WB)
β-actin	Abcam	ab6276	1:1000(WB)
Synaptotagmin(Syt)	Abclonal	A0992	1:1000(WB)
Synapsin1 (Syn1)	Abclonal	A17362	1:1000(WB)
PSD95	Abclonal	A0131	1:1000(WB)
GluA1	Cell Signaling	13185	1:1000(WB)
GluA2	Cell Signaling	13607	1:1000(WB)
GluN2A	Cell Signaling	4205	1:1000(WB)
GluN2B	Cell Signaling	14544	1:1000(WB)
Lamin B1	Abcam	ab16048	1:1000(WB)
STAT1	Cell Signaling	9172	1:1000(WB)
STAT1 (Phospho-Tyr701)	Cell Signaling	9167S	1:1000(WB)
STAT3	Cell Signaling	9139	1:1000(WB)
STAT3 (Phospho-Tyr705)	Cell Signaling	9131	1:1000(WB)

JAK1	Cell Signaling	3344S	1:1000(WB)
JAK1 (Phospho-Tyr1034)	Cell Signaling	74129S	1:1000(WB)
NF-κB	Proteintech	10745-1-AP	1:1000(WB)
Iba1	Wako	019-19741	1:1000(WB) 1:300(IF)
GFAP	Abcam	ab7260	1:300(IF)
NeuN	Cell Signaling	36662	1:300(IF)
DAPI	Cell Signaling	4083	1:1000(IF)
Alexa Fluor 488 AffiniPure Goat Anti-Mouse IgG	Jackson ImmunoResearch	115-545-003	1:300(IF)
CY3 AffiniPure Goat Anti Rabbit IgG	Jackson ImmunoResearch	111-165-003	1:300(IF)
